# Thermochemical Route for Extraction and Recycling of Critical, Strategic and High Value Elements from By-Products and End-of-Life Materials, Part I: Treatment of a Copper By-Product in Air Atmosphere

**DOI:** 10.3390/ma12101625

**Published:** 2019-05-17

**Authors:** Ndue Kanari, Eric Allain, Seit Shallari, Frederic Diot, Sebastien Diliberto, Fabrice Patisson, Jacques Yvon

**Affiliations:** 1 GeoRessources Laboratory, UMR 7359 CNRS, CREGU, Université de Lorraine, 2, rue du doyen Roubault, BP 10162, 54505 Vandoeuvre-lès-Nancy, France; ericgallain@gmail.com (E.A.); frederic.diot@univ-lorraine.fr (F.D.); jacques.yvon@univ-lorraine.fr (J.Y.); 2 Agricultural University of Tirana, Faculty of Agriculture and Environment, 1029 Tirana, Albania; seitshallari@gmail.com; 3 Institut Jean Lamour, UMR 7198 CNRS, Labex DAMAS, Université de Lorraine, Campus Artem, 2 allée André Guinier, BP 50840, 54011 Nancy, France; sebastien.diliberto@univ-lorraine.fr (S.D.); fabrice.patisson@univ-lorraine.fr (F.P.)

**Keywords:** critical and strategic materials, valuable metals, by-products, copper anode slime, thermal treatment, selenium dioxide

## Abstract

Development of our modern society requests a number of critical and strategic elements (platinum group metals, In, Ga, Ge…) and high value added elements (Au, Ag, Se, Te, Ni…) which are often concentrated in by-products during the extraction of base metals (Cu, Pb, Zn…). Further, recycling of end-of-life materials employed in high technology, renewable energy and transport by conventional extractive processes also leads to the concentration of such chemical elements and their compounds in metallurgical by-products and/or co-products. One of these materials, copper anode slime (CAS), derived from a copper electrolytic refining factory, was used for this study. The sample was subjected to isothermal treatment from 225 to 770 °C under air atmosphere and the reaction products were systematically analyzed by scanning electron microscopy through energy dispersive spectroscopy (SEM-EDS) and X-ray diffraction (XRD) to investigate the thermal behavior of the treated sample. The main components of the anode slime (CuAgSe, Cu_2-x_Se_y_S_1-y_, Ag_3_AuSe_2_) react with oxygen, producing mostly copper and selenium oxides as well as Ag-Au alloys as final products at temperatures higher than 500 °C. Selenium dioxide (SeO_2_) is volatilized and recovered in pure state by cooling the gaseous phase, whilst copper(II) oxide, silver, gold and tellurium remain in the treatment residue.

## 1. Introduction

Nowadays, besides the most common metals (Fe, Al, Cu, Zn, Ni…), a wide range of chemical elements (metals, non-metals, and metalloids) are in increasing demand and crucial for the development of renewable energies, the manufacture of electrical and electronic equipment, transportation, advanced industries, which constitute the foundation of innovative high technologies. Often suffering from a slight subjective and arbitrary classification, these elements known as rare elements, or sometimes as small, green, or high-tech metals, are commonly considered to be critical and strategic materials. According to criticality assessments, the number of critical materials (CRMs) for the European Union has grown from 14 CRMs in 2011 to 20 in 2014 and was augmented thereafter to reach 27 CRMs in 2017 [1].

While the importance of CRMs in our society and their inventory is clear, the fragility of their supply represents a real challenge for the French, European and global economies. In general, material savings, substitution and recycling (including eco-design) can mitigate the risk of anticipated shortage of critical metals. Critical and strategic materials are often used in unique applications with limited alternatives and substitutions [2]. Note that the actual and future demand for several of these materials are expected to strongly grow, especially for the production of electric and electronic devices, while the life-spans of many products such as computers and cell phone are decreasing rapidly [3]. This phenomenon, among others, leads to the generation of a huge waste stream (waste electrical and electronic equipment-WEEE), often designated as “e-waste” [4,5,6,7]. Its elevated contents in high added values elements (precious, strategic, critical, rare and rare earths elements) make this waste stream a very attractive “urban mine” for the extraction of these components.

But paradoxically, the rate of recycling of CRMs from end-of-life materials is low compared to that of base metals. The presence of several metals of different chemical nature in increasingly miniaturized products, and incorporated in complex matrixes encompassing ceramics, resins, plastics, and especially halogenated substances (based on chlorine and bromine) makes their recycling process quite complex. A spectrum of contributions focused on current state-of-the-art research on metal recycling from wastes was introduced in a special issue (Valuable Metal Recycling) of *Metals* [8]. An extensive overview for the recovery of high value metals from electronic waste and spent catalysts using improved pyrometallurgical and hydrometallurgical technologies was given by Ding et al. [9]. It was mentioned that smelting is beneficial to segregate and concentrate the precious metals from e-waste in the by-products. In a recent investigation, Avarmaa et al. [10] demonstrated that during smelting, copper acts as collector metal for gold, silver, palladium and platinum contained in the end-of-life electronic devices. One may emphasize that most of the targeted elements (Au, Ag, Se, Te, platinum group metals, etc.) are recovered in anode slime from primary copper extraction from its sulfide ores and concentrates. In summary, recycling of ‘e-waste’ by pyrometallurgical route in the existing copper metallurgy leads to the concentration of these elements in by-and/or co-products, mostly in the anode slime of copper electrorefining.

A number of research reports were devoted to extraction and recovery of specific elements from copper anode slime (CAS). The most typical investigations concerned CAS treatment by H_2_SO_4_-O_2_ to recover Cu followed by thiourea leaching for Ag extraction [11] or leaching in a sulfuric and nitric acid medium which was used for silver nanoparticles synthesis [12]. A nitric-sulfuric acid mixture was also tested by Li et al. [13] for the recovery of Cu, Ag and Se from a CAS sample, while Xiao et al. [14] reported an oxidative pretreatment by thiosulfate leaching followed by an electrodeposition process for the selective recovery of Ag. Dissolution of selenium in NaOH, after sulfuric acid decopperization of CAS, was also reported [15]. However, little recent information is available for the thermal behavior of CAS under an oxidizing atmosphere. In this frame, the work presented in this article deals with the treatment in an air atmosphere of a CAS sample generated by a European copper plant. Particular attention was payed to the speciation and physical characterization of the obtained products (residues and condensates) at various temperatures to determine the reaction mechanisms and to identify process steps for a possible selective separation of the CAS components.

This research work is an extension of our investigations on the concentration and extraction of a broad range of valuable metals from raw materials and solid industrial residues by thermal route [16,17,18,19,20] as well as on the recycling and valorization of co-products and wastes via chemical synthesis of new materials [21,22,23,24].

At least two of the researched elements of this investigation, selenium and tellurium, belong also to the scattered elements category; i.e., which exist at low content, in a decentralized state and rarely form independent minerals in nature [25]. Both elements are used in thin films (CIGS—copper indium gallium selenide and CdTe—cadmium telluride) and used in second-generation modules of photovoltaic panels [26,27,28]. Efficient extraction and recycling of these elements from by-products, end-of-life solar photovoltaics and other wasted materials will be a main research focus in the future to meet the volume of industrial demand.

## 2. Materials and Methods

A sample of copper anode slime provided from a refining factory available as a powdered solid with a mean particles size less than 30 µm was used for this study. The sample composition was determined by diverse analytical methods, but only the results of scanning electron microscopy-energy dispersive spectroscopy (SEM-EDS) and X-ray diffraction (XRD) analyses were selected to be presented in this paper (see Section 3). SEM examinations were performed by using a HITACHI S-4800 device (Hitachi Ltd., Tokyo, Japan) equipped with an EDS elemental analysis microprobe. SEM imaging and chemical microanalysis were performed mostly at an accelerating voltage of 15 kV. The samples were carbon coated prior to analysis. XRD was performed on samples using a Bruker D8 Advance device (Bruker, Karlsruhe, Germany), (Co Kα1 radiation, λ = 1.789 Å), under 35 kV and 45 mA operating conditions. The acquisition of diffraction patterns was performed between 3° and 64° (2θ) range at step scan 0.034° with 3 s per step. The XRD-patterns were analyzed thanks to the DIFFRAC.EVA software (version 5, Bruker) and PDF-2 release 2011 database.

Isothermal experimental tests for the CAS sample treatment were conducted in horizontal set-ups, including a system of static tubular furnaces that were able to reach 1600 °C. To achieve this, a pre-weighted sample of several grams was introduced directly into the furnace preheated at the desired temperature. When the dwell time was reached, the sample was removed from the furnace and cooled down to room temperature. Air was used as a flowing gas and it also performed the oxidation of CAS components. The outlet gases were cooled at room temperature, leading to the condensation of the vapor phase and the recovery of a solid condensate. Initial CAS sample and solid products obtained from CAS thermal treatment were examined by visible microscopy, SEM-EDS and XRD. The advantage of using SEM-EDS analysis here is the ability to gather punctual information about elemental content, phases differentiation, and about the morphological and textural evolution of the thermally treated samples, all this contributes to a better understanding of the involved reaction mechanism and processing steps.

## 3. Results

### 3.1. Elemental and Mineralogical Analysis of CAS Sample

**Figure 1:**
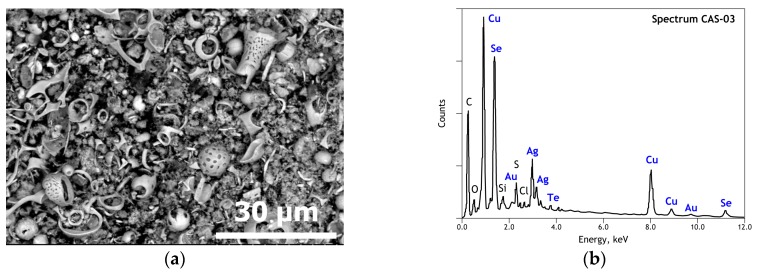
SEM-EDS results of initial copper anode slime: (**a**) General view (backscattered electron micrograph) of the used sample; (**b**) Overall EDS analysis of the used sample.

Several research reports [11,12,13,14,15,29,30,31,32] found in the literature revealed that the copper anode slime is a quite complex material in elemental and mineralogical composition, as well as in morphological aspects. This complexity resulted from the nature of copper raw materials, operating conditions of copper extractive processes (smelting, converting, fire- and electro-refining) and from the decopperization step applied to treat CAS. By the same token, a general SEM image of the used CAS sample for this study given in Figure 1a shows a particular morphology of this solid. Besides the spherical particles, there are many particles that are irregularly and shell shaped. As clearly shown by the SEM image, most particles are corroded, resulting in three-dimensional lens-like forms and other ones with plenty of cavities and pores.

The general EDS spectrum of the CAS (Figure 1b) indicated a multi-elemental composition with a descending peak intensity order as follows: Cu, Se, Ag, O, S, Au, Te, Si, Cl. There is probably also a carbonaceous matter in the CAS sample, but it was difficult to distinguish from the carbon coating used to make the sample conductive before SEM-EDS analysis.

**Figure 2:**
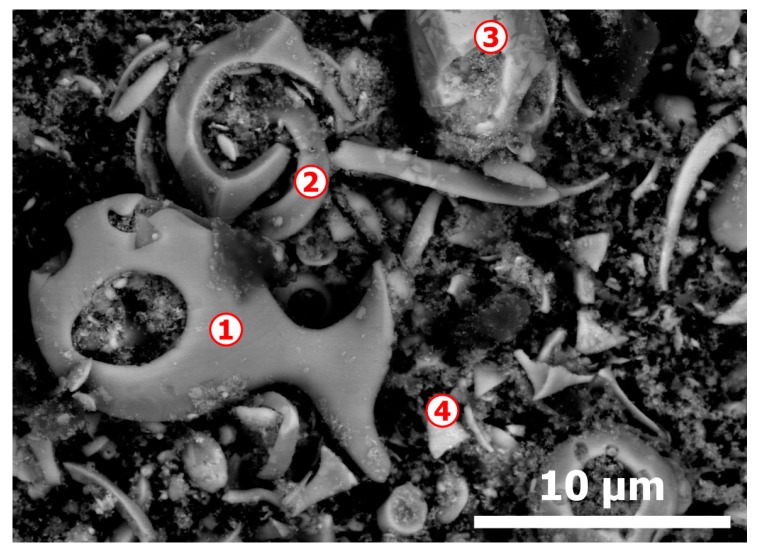
Detailed aspects (backscattered electron micrograph) of initial CAS as revealed by SEM-EDS.

The morphology of the CAS sample showed in Figure 2 and SEM-EDS microanalysis data summarized in Table 1, indicates that there are particles containing mostly Cu-Se (spot n° 1 and 2), Ag-Se-Cu-Te (spot n° 3) and Ag-Se-Cu-Au-Te (spot n° 4); gold is found mostly in the finest particles. There is also some sulfur in almost all particles, while the small amount of oxygen is bound to silicon. This peculiar state of CAS particles together with their composition can be explained by the preceding impact of at least two factors: (i) the behavior the Cu, Ag, Au, Se, Te, S, O and others elements compounds in fire refining process of blister copper and (ii) the chemical and electrochemical reactivity of these compounds towards electrolyte during electrorefining of anode copper. The fire refining process, with the goal of removing residual oxygen and sulfur as well as other impurities from blister copper before casting into anode, is achieved at 1200–1250 °C.

As could be expected, the residual oxygen and sulfur can be associated with copper forms as Cu_2_O and Cu_2_S, having higher melting points than copper. One may deduce that during the cooling of casted Cu-anodes, the Cu_2_O and Cu_2_S particles, which are first to be solidified, can serve as heterogeneous nuclei for further solidification around these nucleation sites. The phase diagrams for copper with chalcogen (e.g., S, Se, or Te) show that there are miscibility gaps in the liquid phase [33] that will allow the solidification of droplets with the highest melting points followed by further solidification of low melting point components, thereby resulting in a layered structure and in a significant difference between the core and the periphery of the obtained solid. During copper electrorefining, the copper oxide (CuO) contained in mixed (Cu, Se, Te, S, O…) particles as well as common impurities (most probably oxides of As, Sb and Bi) are dissolved, besides electrochemical dissolution of Cu°, resulting in the final configuration of CAS. These observations are in good agreement with other research works reported in the literature [29,30,31,32].

**Figure 3:**
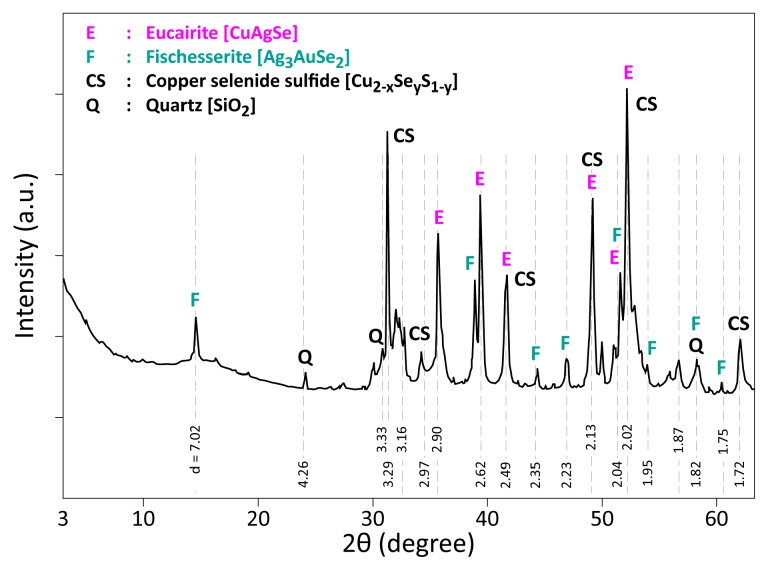
XRD patterns of CAS raw sample.

XRD results of the used CAS are shown in Figure 3. The main crystallized phase is Eucairite (CuAgSe), confirming the microanalysis obtained by SEM-EDS analysis. However, this phase probably forms during the electro-refining process. According to Chen and Dutrizac [29,32], silver is in a metastable state in the copper anode and this can promote the chemical and/or electrochemical dissolution of Ag° during electrorefining. The resulting ion Ag^+^ can react following Equation (1) by synthetizing the Eucairite phase. In addition, the authors stated that the particles kept the original morphologies of Cu_2_Se [Cu_2_(Se,Te)], although their composition has been changed. The conditions of the electrorefining process (mean values: T ≈ 60–65 ° C; ≈ 180–185 g/L H_2_SO_4_; residence time of several days) will certainly favor such a reaction.

Ag^+^ + Cu_2_(Se,Te) → AgCu(Se,Te) + Cu^+^ (1)

In the XRD patterns of CAS, there are some peaks corresponding to Cu_2_Se, Cu_2_S, CuSeS, as well as to non-stoichiometric ones with an absence of certain peaks due probably to crystallite orientation; for simplification, all of these phases are designated as copper selenide sulfide (Cu_2−x_Se_y_S_1−y_). Regarding the Au-bearing phase, the XRD peaks of the obtained diffractogram match most frequently with Fischesserite (Ag_3_AuSe_2_). This phase composition (with substitution of Ag by Cu and Se by Te) is also confirmed by SEM-EDS analysis (spot n° 3 and spot n° 4 in Table 1). Finally, Quartz (SiO_2_) completes the set of crystallized phases identified in the CAS sample.

### 3.2. Thermal Treatment of CAS in Air for 1 h

**Figure 4:**
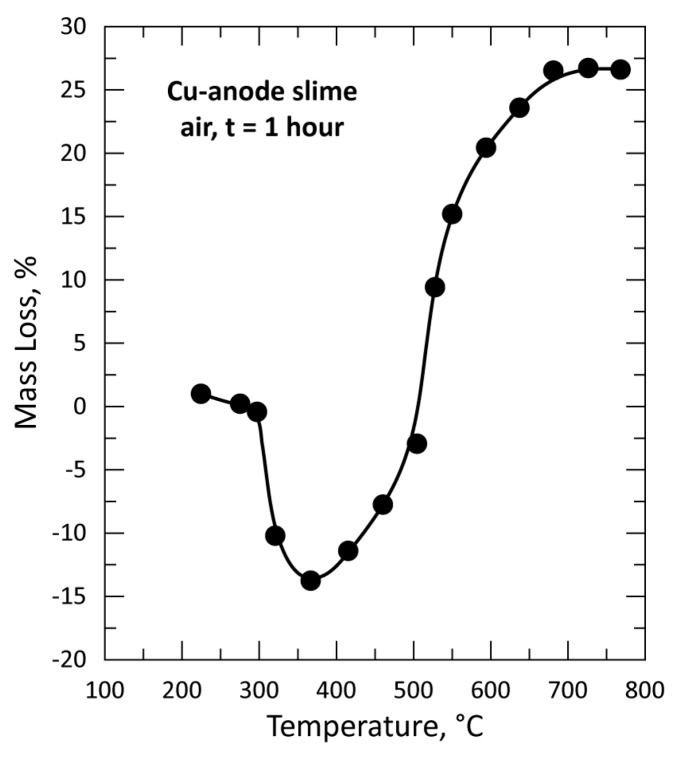
Evolution of the mass loss of the sample versus temperature during treatment of CAS in air for 1 h.

Several grams of the copper anode sample were used for isothermal tests performed between 225 °C and 770 °C in air atmosphere with a flow rate of 25 L/h. The evolution of the % mass loss (%ML) as a function of temperature is plotted in Figure 4. The %ML curve shape suggests that many reactions steps may occur in the explored temperature interval. The sample started to react with air oxygen (O_2_) at temperatures higher than 225 °C, resulting in a mass gain of up to about 350 °C. From here, there is a continued mass loss up to around 700 °C, where the mass loss is stabilized.

According to the composition of raw CAS sample, the main constituents which may strongly affect the %ML are Cu, Se and Ag combined as selenides of copper and silver. During their reactions with air (oxide formation) and knowing the atomic mass of these elements (Cu: 63.55; Se: 78.97 and Ag: 107.87, O: 16.00), it appears that no volatilization of these elements and/or their oxides occurred, at least up to 370 °C, since only a mass gain was recorded. The most volatile compound of the Cu-Se-Ag-O system is SeO_2,_ having a vapor pressure of 73 kPa at 320 °C [34] which is enough for its volatilization during the CAS treatment at this temperature. This is an indirect confirmation that no free SeO_2_ is formed at T < 320 °C when CAS sample is heated in air. More consistent information about the interaction steps of CAS components with air is given in the subsequent section.

### 3.3. Analysis of the Reaction Products

**Figure 5:**
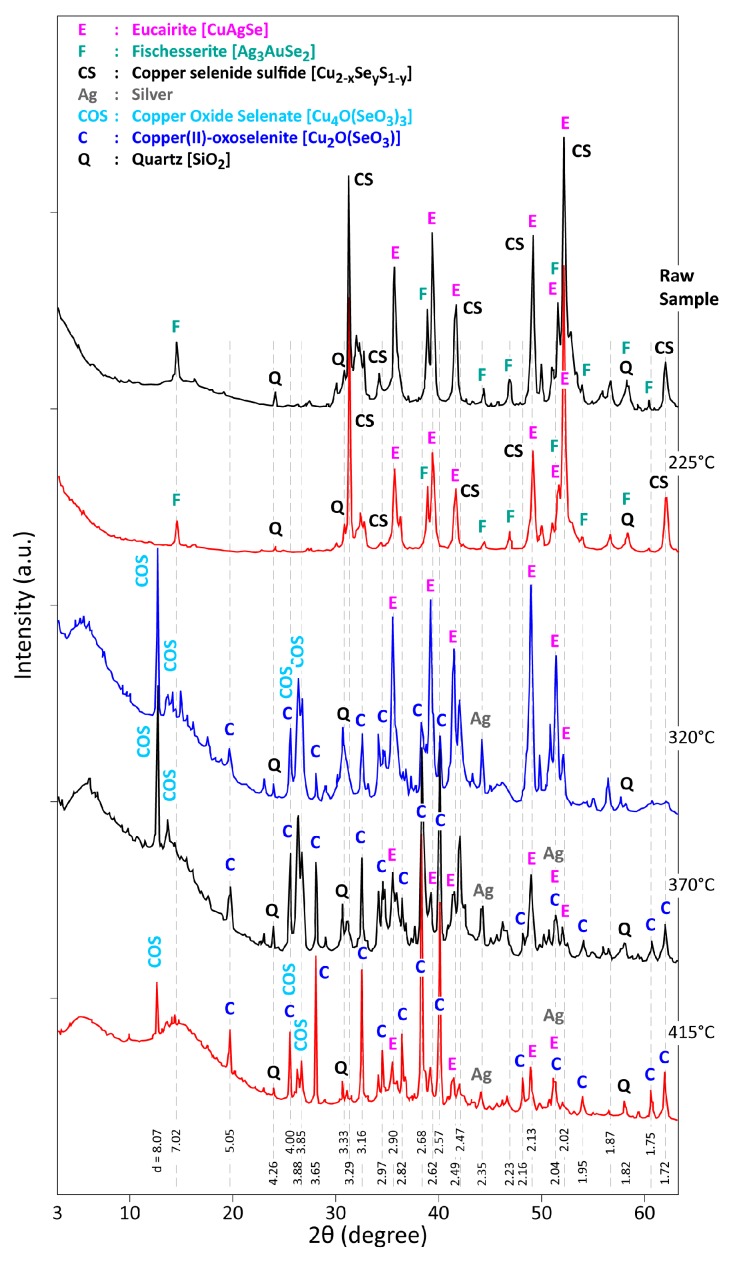
XRD patterns of CAS raw sample and the residues obtained during CAS sample treatment under air, from 225 °C to 415 °C.

**Figure 6:**
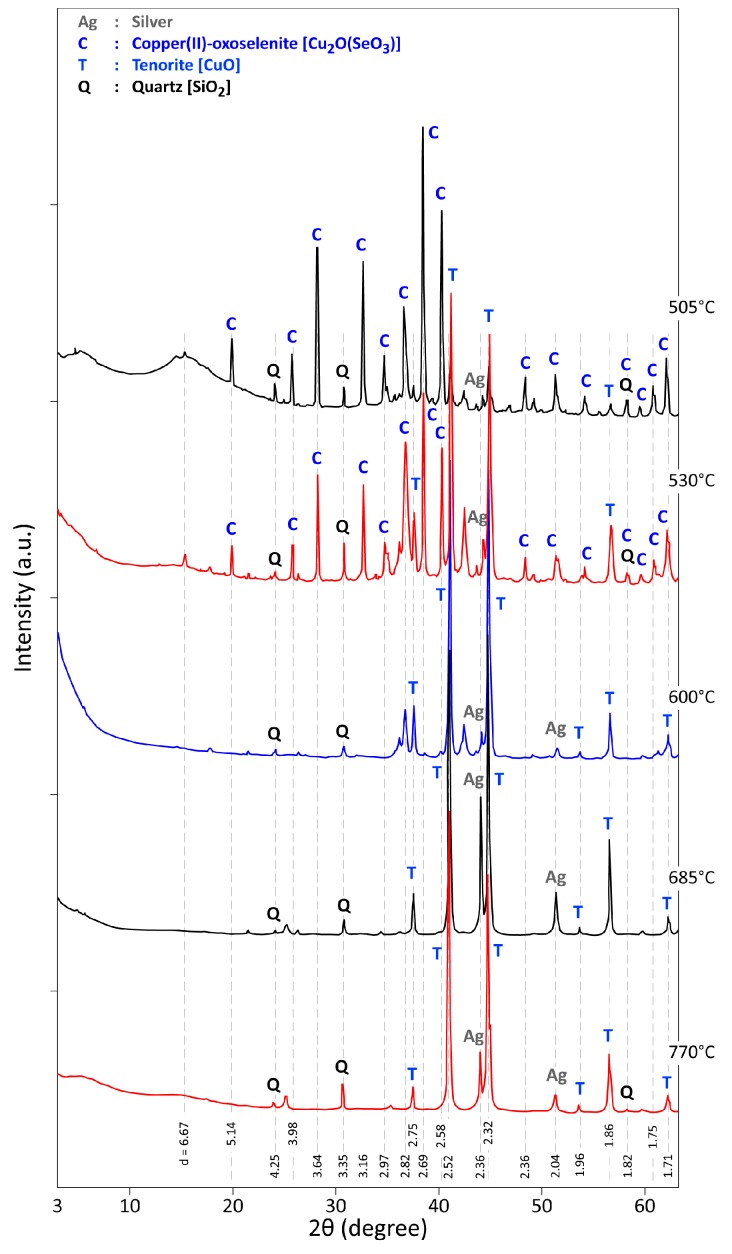
XRD patterns of the residues obtained during CAS sample treatment under air from 505 °C to 770 °C.

The treatment residues issued from isothermal tests of CAS under air atmosphere were systematically examined by XRD technique and results are presented in Figure 5 and Figure 6 for the treatment temperatures of 225–415 °C and 505–770 °C, respectively.

As shown in Figure 5, the X-ray diffractogram of the residue obtained at 225 °C is similar to that of the CAS raw sample. The XRD patterns of the sample heated 320 °C exhibits a fair broad diffraction band near to 6° (2θ), indicating a slight XRD disordered structure, at least, that of a reaction product which will be a precursor of further crystallized phases. Eucairite (CuAgSe) seems to be still stable at these temperatures, while the characteristic peaks of Cu_2−x_Se_y_S_1−y_ and Ag_3_AuSe_2_ phases disappeared. Apparition of new crystallized phases such as: [Cu_4_O(SeO_3_)_3_], [Cu_2_O(SeO_3_)] and Ag° is obvious from the XRD examination. One may hypothesize that [Cu_4_O(SeO_3_)_3_], which can be written also as [4CuO·3SeO_2_], is the first crystallized phase issued from the reaction of copper selenide bearing substances with O_2_, though other phases not detected by XRD may be the intermediate product of selenide oxidation by oxygen. Metallic silver is also identified in this treatment residue; note that the main XRD peaks of Ag° and Au° overlapped. Further increase in the treatment temperature (from 320 °C to 415 °C) of CAS sample leads to a decrease in the reflection intensities of CuAgSe and Cu_4_O(SeO_3_)_3_ in the generated residue, while the relative intensity of [Cu_2_O(SeO_3_)] peaks seems increased. As noted by Portnichenko et al. [35], copper(II)-oxoselenite [Cu_2_OSeO_3_] has a cubic structure with a cell parameter a = 8.925 Å.

At 505 °C (Figure 6), [Cu_2_O(SeO_3_)] is still a main crystallized phase, but a new phase, tenorite (CuO) is also identified and becomes the predominant phase at 600 °C. Based on the %ML of Figure 4, on the results of XRD diffraction and data from the research work performed by Fokina et al. [36], the two steps of Cu_4_O(SeO_3_)_3_ conversion into CuO and SeO_2_ as final products can be described by Equations (2) and (3). Diffractograms of products obtained at 685 °C and 770 °C confirm the presence of well-crystallized phases of silver (gold), tenorite and quartz, which was present in all analyzed residues. Though some sulfur is present in the CAS sample, often substituted by selenium and/or tellurium, no solid phase containing sulfur was identified in the thermal treatment residue.

Cu_4_O(SeO_3_)_3_ → 2Cu_2_O(SeO_3_) + SeO_2_ (2)

2Cu_2_O(SeO_3_) → 4CuO + 2SeO_2_ (3)

**Figure 7:**
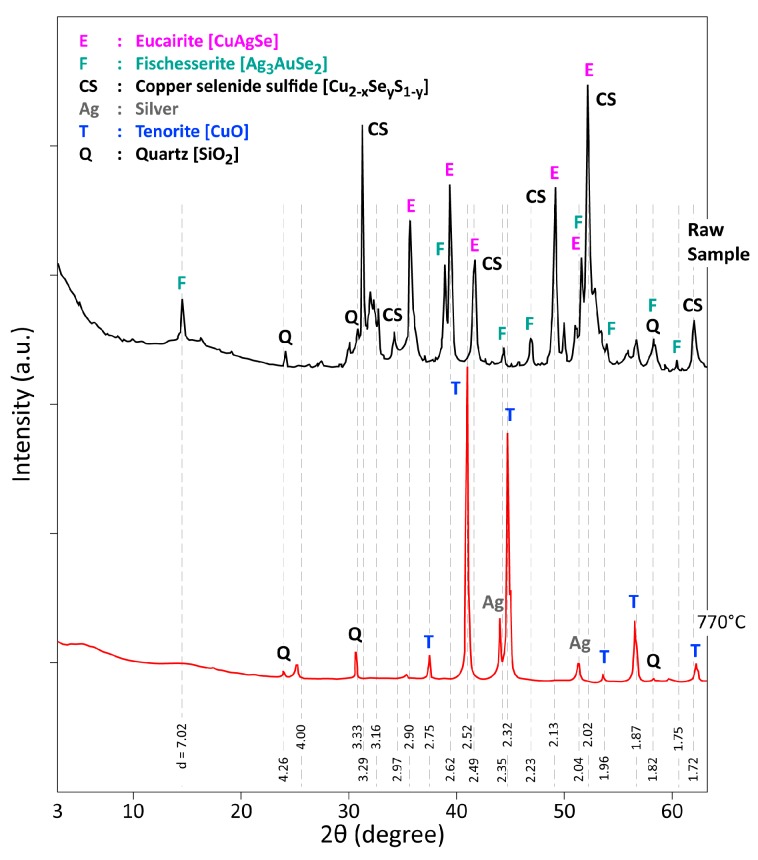
XRD patterns of a CAS raw sample and its treatment product in air at 770 °C.

One may emphasize that no complete and systematic data of XRD analysis were found in the literature for monitoring the behavior of such copper anode slime under air in a wide temperature interval (225–770 °C). A comparison of XRD patterns of a CAS raw sample and its reaction product resulting from the thermal treatment of CAS at 770 °C in air is given in Figure 7. In summary, with a complex phase composition of a raw CAS sample (Eucairite-CuAgSe; copper selenide sulphide-Cu_2−x_Se_y_S_1−y_; Fischesserite-Ag_3_AuSe_2_ and Quartz-SiO_2_), the treatment residue at 770 °C is free of selenium bearing compounds and it is composed of cupric oxide, quartz and silver (gold). These compounds are concentrated in the residue as the thermal treatment allowed the volatilization of selenium dioxide, leading to a mass loss of sample exceeding 25 percent.

**Figure 8:**
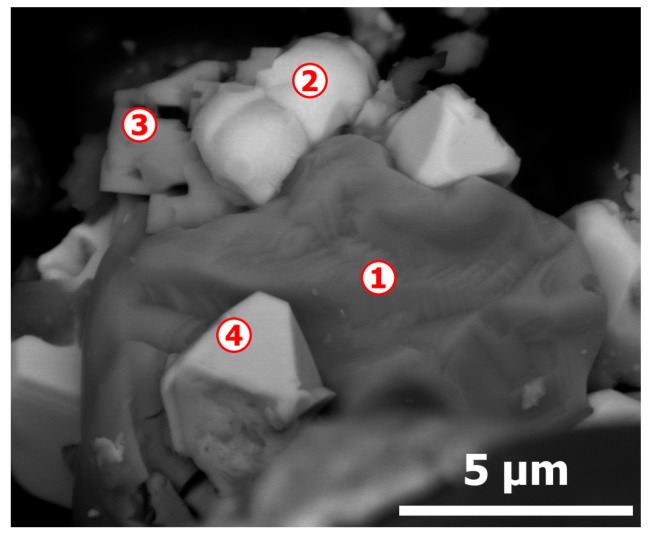
SEM aspects (backscattered electron micrograph) of the CAS sample treated at 770 °C in air.

Likewise, several residues were examined by SEM-EDS technique. A typical morphology is given in Figure 8 with a visible difference in contrast suggesting the presence of areas of different compositions. Data of EDX analysis of spots 1 through 4 are summarized in Table 2. Within the measurement errors, the particle n° 1 is essentially composed of CuO (tenorite). The EDS analysis of spot n° 2 showed that silver (c.a., 78.6 wt %) is the major constituent with some gold (c.a., 14.6 wt %) and copper (c.a., 4.6 wt %). A possible zonal fusion may explain the smoothed shape of this particle. Copper and tellurium oxide (TeO_2_) seem to be the main components of the area noted as n° 3. The composition of crystal-like n° 4 is similar of spot n° 2 with a quite different morphology. Note that selenium is not detected in the treatment residue. These data seem to confirm those obtained by XRD diffraction analysis.

**Figure 9:**
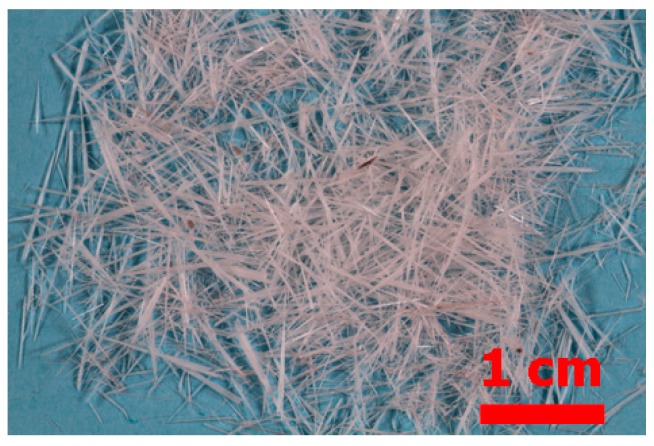
Visible microscopy image of the solid condensate obtained during CAS treatment at 685°C.

**Figure 10:**
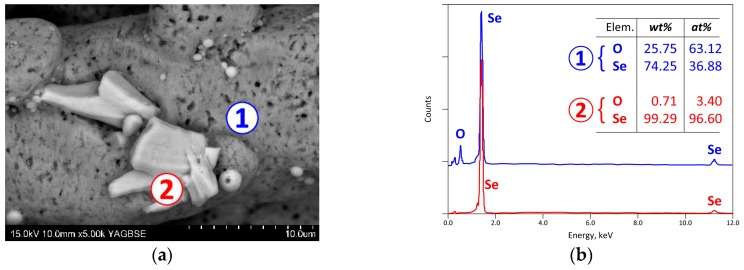
SEM-EDS results of a condensate obtained during thermal treatment of the Cu-anode slime sample: (**a**) General view (backscattered electron micrograph) of SEM with two different phases; (**b**) EDS analysis of the same phases.

During the treatment of CAS at temperatures above 500 °C, a solid mass is condensed in the tepid inner side of the experimental reactor. The visually observed quantity of this solid increased with the reaction progress and the rising temperature. A visible microscopy image of the recovered condensate is given in Figure 9 where centimetric needle-like crystals are observed. The SEM-EDS examination (Figure 10) showed that the solid is homogenous, nevertheless some small zones of spherical and other shapes characterized by micronic sizes were revealed. As shown by Figure 10b, the matrix area of SEM image (Figure 10a) is composed especially of Se and O in atomic proportion close to the SeO_2_ stoichiometry. In contrast, the lighter areas (Spot n° 2 in micrograph of Figure 10a) have a high content of selenium. Reduction of SeO_2_ to Se° is probably achieved by sulfur dioxide (SO_2_) during cooling of the gas phase. Taking into account that the crystallized phases synthesized during the oxidation of CAS by air (Figure 5 and Figure 6) were [Cu_4_O(SeO_3_)_3_] and [Cu_2_O(SeO_3_)], the selenium dioxide (SeO_2_) which is generated from the decomposition of these compounds according to Equations (2) and (3) can be separated by volatilization and recovered in solid state.

This study encompassed some characteristics for the thermal treatment of a copper by-product and allows understanding of the behavior of selected element compounds during the process. However, the end-of-life materials (e-wastes, thin films of used photovoltaics, spent catalysts, wasted flat screen…) containing rare, critical, strategic and high value elements present also a drawback, which is the presence of plastic materials. In addition, these plastics contain a considerable amount of halogenated substances based on chlorine (polyvinyl chloride-PVC) and bromine (Br-flame retardants combined with based Sb-synergist). The thermodynamic and kinetic reactivity of these substances with respect to targeted metals during an envisaged thermal process will be the subject of the second part of this investigation.

## 4. Conclusions

With grown in demand of current and future development of new technologies for rare, critical and strategic elements and facing the depletion of basic primary resources, the targeted element extraction and recycling from by-products, wastes and end-of-life materials is a promising choice. Thermal treatment under an oxidizing atmosphere up to 770 °C of the copper anode slime (CAS), a potential source of these elements, led us to reach the following conclusions:

Selenium is mostly found in the copper-silver bearing phase (CuAgSe) and/or bounded to copper. Tellurium and sulfur can substitute selenium in the phase formulation. Gold is found in finest particles and Ag_3_AuSe_2_ seems to be the most likely gold bearing phase.

The thermal treatment of CAS at low temperatures led to the formation of double Cu-Se oxide [Cu_4_O(SeO_3_)_3_] which decomposed at temperatures higher than 400 °C.

Copper(II)-oxoselenite [Cu_2_O(SeO_3_)], synthetized also at low temperatures, is stable, at least up to 530 °C. At higher temperatures, only tenorite (CuO) is found in the treatment residues as a decomposition product of these double oxides, while selenium dioxide is volatilized.

Almost pure SeO_2_ is recovered by condensation of outlet gases issued from thermal treatment of CAS at temperatures higher than 500 °C.

The final residue of the CAS treatment under air beyond 600 °C is mostly composed of CuO and alloys of Ag-Au (with some Cu°), while tellurium is found as oxide (TeO_2_), and silica (SiO_2_) remains intact. Further treatment of such material is required for the final separation of these high value element compounds.

## Figures and Tables

**Table 1:** Elemental composition of initial CAS analyzed by SEM-EDS.

Elements	Spot n° 1		Spot n° 2		Spot n° 3		Spot n° 4	
	wt % ^1^	at % ^1^	wt %	at %	wt %	at %	wt %	at %
O	-	-	-	-	-	-	0.49	2.55
Si	-	-	-	-	-	-	1.01	3.00
S	2.01	4.12	2.87	6.01	1.72	4.36	1.46	3.79
Cl	-	-	-	-	0.63	1.45	-	-
Cu	72.20	74.48	58.55	61.88	22.21	28.47	30.79	40.28
Se	25.78	21.40	35.48	30.17	32.57	33.59	20.34	21.42
Ag	-	-	3.11	1.93	40.88	30.89	27.05	20.84
Te	-	-	-	-	1.98	1.27	0.67	0.43
Au	-	-	-	-	-	-	18.19	7.68

^1^ wt % and at % represent mass and atomic percentage, respectively.

**Table 2:** Elemental composition (EDS data) of the CAS treated at 770 °C in air atmosphere.

Elements	Spot n° 1		Spot n° 2		Spot n° 3		Spot n° 4	
	wt % ^1^	at % ^1^	wt %	at %	wt %	at %	wt %	at %
O	18.35	46.66	1.48	9.34	15.35	47.05	-	-
Al	0.59	0.89	-	-	0.67	1.22	-	-
Si	0.67	0.97	0.72	2.57	1.13	1.97	-	-
Cu	80.39	51.48	4.56	7.22	45.96	35.47	4.38	7.80
Ag	-	-	78.61	73.39	1.57	0.71	78.56	82.40
Au	-	-	14.62	7.48	-	-	17.06	9.80
Te	-	-	-	-	35.33	13.58	-	-

^1^ w% and at % represents mass and atomic percentage, respectively.
